# Local and Long-Range Circuit Connections to Hilar Mossy Cells in the Dentate Gyrus

**DOI:** 10.1523/ENEURO.0097-17.2017

**Published:** 2017-04-19

**Authors:** Yanjun Sun, Steven F. Grieco, Todd C. Holmes, Xiangmin Xu

**Affiliations:** 1 Department of Anatomy and Neurobiology, School of Medicine, University of California, Irvine, CA 92697-1275; 2 Department of Physiology and Biophysics, School of Medicine, University of California, Irvine, CA 92697-4560; 3 Department of Biomedical Engineering, University of California, Irvine, CA 92697-2715; 4 Department of Microbiology and Molecular Genetics, University of California, Irvine, CA 92697-4025

**Keywords:** hippocampus, excitatory input, inhibitory input, synaptic connections, viral tracing

## Abstract

Hilar mossy cells are the prominent glutamatergic cell type in the dentate hilus of the dentate gyrus (DG); they have been proposed to have critical roles in the DG network. To better understand how mossy cells contribute to DG function, we have applied new viral genetic and functional circuit mapping approaches to quantitatively map and compare local and long-range circuit connections of mossy cells and dentate granule cells in the mouse. The great majority of inputs to mossy cells consist of two parallel inputs from within the DG: an excitatory input pathway from dentate granule cells and an inhibitory input pathway from local DG inhibitory neurons. Mossy cells also receive a moderate degree of excitatory and inhibitory CA3 input from proximal CA3 subfields. Long range inputs to mossy cells are numerically sparse, and they are only identified readily from the medial septum and the septofimbrial nucleus. In comparison, dentate granule cells receive most of their inputs from the entorhinal cortex. The granule cells receive significant synaptic inputs from the hilus and the medial septum, and they also receive direct inputs from both distal and proximal CA3 subfields, which has been underdescribed in the existing literature. Our slice-based physiological mapping studies further supported the identified circuit connections of mossy cells and granule cells. Together, our data suggest that hilar mossy cells are major local circuit integrators and they exert modulation of the activity of dentate granule cells as well as the CA3 region through “back-projection” pathways.

## Significance Statement

Hilar mossy cells are the neuron type of considerable interest in the dentate hilus. However, due to the technical difficulty of targeting mossy cells for *in vivo* circuit mapping, many aspects of local and long-range synaptic connections to these neurons remain uncharacterized. In this study, we used novel viral-genetic tracing and functional circuit mapping approaches to map and compare large-scale circuit connections to hilar mossy cells and dentate granule cells. We uncover previously unidentified circuits to hilar mossy cells and dentate granule cells. Our data support the proposal that hilar mossy cells function as major local circuit integrators of the dentate gyrus.

## Introduction

The dentate gyrus (DG) is a critical structure within the hippocampal formation and is considered the first stage of information processing in the excitatory tri-synaptic circuitry of the hippocampus (Amaral et al., 2007; Witter, 2007). The excitatory neuronal types in the DG include the much-studied dentate granule cells in the fascia dentata and the mossy cells in the hilus. Hilar mossy cells are the principal and only glutamatergic neurons in the dentate hilus. They were named after their “mossy” appearance due to their relatively large somata and thick bushy proximal dendrites covered by numerous large and complex spines which are the sites of mossy fiber input synapses (Amaral, 1978). Mossy cells receive much attention because of their potentially critical roles in cognition, and their vulnerability to excitotoxicity in temporal lobe epileptogenesis (Scharfman, 2007; Myers and Scharfman, 2011). Early studies of the axon projections of intracellularly labeled mossy cells *in vivo* indicate the axon arbors of single mossy cells extend to both local and distant regions of the hippocampus (Buckmaster et al., 1992; Buckmaster et al., 1996). Most of the axon terminals are concentrated in the DG molecular layer, primarily innervating the dendrites of granule cells. The “granule cell association” hypothesis states that mossy cells integrate inputs from local granule cells and distribute that information to distant granule cells, for associative memory (Buckmaster and Schwartzkroin, 1994; Scharfman and Myers, 2012). Mossy cells have also been proposed to have an important role in mediating CA3 “back projection” to the DG by relaying excitatory input from CA3 to granule cells (Scharfman, 2007). In addition, there are long range GABAergic and cholinergic septal inputs to the DG, potentially innervating hilar mossy cells (Buckmaster and Schwartzkroin, 1994; Vivar et al., 2012). Thus, mossy cells appear to be well positioned to enhance DG function by integrating intrahippocampal inputs and other modulatory inputs.

Compared with dentate granule cells, mossy cells do not form recognizable layers of densely packed somata, and they are scattered in the hilar region under the granule cell layer. Partly due to the technical difficulty of targeting mossy cells for *in vivo* circuit mapping and their lack of ordered ultrastructure, many aspects of local and long-range circuit inputs to these neurons remain uncharacterized (Scharfman, 2007). To better understand how mossy cells interact with dentate granule cells and other neuronal types to modulate functional circuit operations of the DG, we applied new viral genetic and functional circuit mapping approaches (Wickersham et al., 2007; Gradinaru et al., 2010; Vivar et al., 2012; Kuhlman et al., 2013; Shi et al., 2014; Sun et al., 2014) to quantitatively map and compare local and long-range circuit connections of mossy cells and dentate granule cells. We combined selective viral genetic systems with monosynaptic rabies retrograde tracing of synaptic connections to uncover previously unidentified circuits to hilar mossy cells and dentate granule cells. These findings provided a new view of information flow through these cells. We then functionally confirmed these new connectional features of hilar mossy cell and dentate granule cells with photostimulation-based circuit mapping via fast voltage-sensitive dye (VSD) imaging and whole cell recordings in brain slices. Together, the anatomical and functional evidence reveals that hilar mossy cells are a major locus of local circuit input integration in the DG that exert modulation of activities of dentate granule cells as well as the CA3 region.

## Materials and Methods

### Animals

All experiments were conducted according to the National Institutes of Health guidelines for animal care and use and were approved by the Institutional Animal Care and Use Committee of the University of California, Irvine (UCI). Although the genetically modified rabies viruses used for the proposed experiments are deletion-mutant rabies and are based on a vaccine strain (SAD-B19), they still pose a limited potential health risk with the helper virus. All personnel working with this rabies strain are therefore vaccinated and experiments are conducted under biosafety level 2 conditions with a protocol approved by the UCI institutional biosafety committee.

In the viral circuit tracing experiments, wild-type C57BL/6J mice (The Jackson Laboratory) were used to map the input connections of hilar mossy cells, while D1-Cre transgenic mice (Lemberger et al., 2007) were used to map the input of dentate granule cells. Mice 8-12 weeks old (either sex) were used for experiments, and they had free access to food and water in their home-cages before and after surgeries. For fast VSD imaging experiments, C57BL/6J mice (either sex) at the age of three weeks were used for imaging. For laser scanning photostimulation (LSPS) experiments, Gad2-Cre; Ai9 mice (either sex) at the age of four weeks were used. They were obtained from crossing the GAD2-IRES-Cre line (stock number 028867, The Jackson Laboratory) with the Ai9 reporter line (stock number 007905, The Jackson Laboratory).

### Viral injections

To perform stereotaxic viral injections into the brain, mice were anesthetized under 1.5% isoﬂurane with a 0.8 L/min oxygen flow rate using an isoﬂurane table top unit (HME109, Highland Medical Equipment). Mice were then placed in a rodent stereotax (Leica Angle Two for mouse) with continuous 1% isoflurane anesthesia with the head secured. A small incision was made in the head, the skin reflected, and the skull exposed to show the landmarks of bregma and lambda, and the injection sites were located. As part of the Leica Angle Two system, a three-axis micromanipulator guided by a digital atlas was used to determine coordinates for the injection sites. A small drill hole was made in the skull over the injection site, exposing the pia surface. A pulled glass pipette (tip diameter, ≈20 μm) was loaded with viruses and then lowered into the brain with the appropriate coordinates. A Picospritzer (General Valve) was used to pulse the virus into the brain. To specifically target hilar mossy cells in the wild-type mice, 0.5 μl of a WGA-Cre expressing adeno-associated virus (AAV) (AAV2-EF1a-mCherry-IRES-WGA-Cre, ∼1 × 10^12^ genomic copies (GC)/ml, UNC vector core) was injected into one side of the DG (AP -2.06 mm, ML 1.94 mm, DV -2.08 mm; all values given relative to the bregma). Then 0.1 μl of a Cre-dependent AAV helper virus expressing histone-tagged EGFP, TVA950, and B19 glycoprotein (AAV8-EF1a-FLEX-HTB, ∼2 × 10^13^ GC/ml) was injected into the contralateral DG (AP -2.06 mm, ML -1.94 mm, DV -1.97 mm). The viruses were injected into the brain at a rate of 20-30 nL/min, with 10-ms pulse duration. For dentate granule cell cases targeted by using the D1-Cre mice, AAV helper virus (AAV8-EF1a-FLEX-HTB) was delivered into the left DG (AP -2.54 mm, ML -1.95 mm, DV -2.01 mm) through iontophoresis with a positive 3-μA current at 7 s “on” and 7 s “off” cycles for 5 min. To prevent virus backﬂow after the delivery, the pipette remained in the brain for 5 min after completion of the injection. Once the injection pipette was withdrawn, the mouse was removed from the stereotax, and the incision was closed with either wound clips or tissue adhesive (3M Vetbond). Mice then recovered in their home cages. At three weeks after the AAV injection, 0.1 μl of a EnvA pseudotyped, RG-deleted rabies virus (EnvA-SADΔG-mCherry rabies, ∼2 × 10^9^ infectious units/ml) was injected into the same location as the previous AAV injection. The rabies virus was allowed to replicate and retrogradely spread from targeted Cre+ cell types to directly connected presynaptic cells for nine days before the animals were perfused for tissue processing.

### Histology, immunochemical staining, and image data acquisition

The mice were transcardially perfused with 5 ml of PBS, followed by 25-ml PBS containing 4% paraformaldehyde. The brains were removed and left in 4% paraformaldehyde overnight, then transferred into 30% sucrose in PBS the next day. The brain was then sectioned coronally at a 30-μm thickness on a freezing microtome (Leica SM2010R). Every third section was mounted for examination and quantification of the starter cells and their presynaptic cells in different brain structures. As both GFP and mCherry expression was strong in rabies-labeled cells, we did not perform immunostaining against either GFP or mCherry. However, selected sections were immunostained with various antibodies for neurochemical characterization of starter cells and rabies-labeled cells in different regions. Conventional immunochemistry was performed as described previously (Xu et al., 2010b). To identify hilar mossy cells, calretinin (CR) immunostaining was performed with a rabbit anti-CR primary antibody (Swant, 1:500; RRID: AB_2619710) followed with an Alexa Fluor 647-conjugated donkey anti-rabbit secondary antibody (Jackson ImmunoResearch, 1:200 dilution). To immunochemically identify GABAergic cells, GABA immunostaining was performed with a rabbit anti-GABA primary antibody (Sigma-Aldrich, 1:1000; RRID: AB_477652) followed with an Alexa Fluor 488- or Alexa Fluor 647-conjugated donkey anti-rabbit secondary antibody (Jackson ImmunoResearch, 1:200 dilution). To identify cholinergic cells in the medial septum-diagonal band of Broca (MS-DB), choline acetyltransferase (ChAT) immunostaining was used with a goat anti-ChAT primary antibody (Millipore, 1:300; RRID: AB_2079751) followed with an Alexa Fluor 488-conjugated donkey anti-goat secondary antibody (Jackson ImmunoResearch, 1:200). For PV staining, a rabbit anti-PV primary antibody was used (Swant, 1:1000; RRID: AB_2631173) followed with an Alexa Fluor 647-conjugated donkey anti-rabbit secondary antibody (Jackson ImmunoResearch, 1:200). Sections were counterstained with 10 μM DAPI, then mounted and cover-slipped with a Vectashield antifade mounting medium (Vector Laboratories). Using Automated Slide Scanning and Analysis software (MetaMorph) in a high-capacity computer with a fluorescent BX61/BX63 Olympus microscope and a high-sensitive Hamamatsu CCD camera, we were able to obtain sufficient-resolution images suitable for all subsequent computer-based analyses under a 10× objective. Image stitching, overlaying, cell counting and further imaging analysis were completed using the MetaMorph imaging and analysis software or Adobe Photoshop CS4 extended version (Adobe Systems) analysis tools. Quantitative examinations across the series of sections were conducted for unbiased analyses of rabies-mediated, direct synaptic connections to targeted Cre-defined cell types. To acquire high resolution and z-stacked images, we also imaged labeled cells in selected sections with a Carl Zeiss or a Leica confocal laser scanning microscope (LSM 780/700, Carl Zeiss; Leica TCS SP8, Leica Microsystems).

### Living brain slice preparation

C57BL/6J or Gad2-Cre; Ai9 mice were deeply anesthetized with pentobarbital sodium (>100 mg/kg, i.p.) and rapidly decapitated, and their brains were removed. Hippocampal slices 400 µm thick were cut at an angle of 20–30° in the horizontal plane to conserve the intrahippocampal axonal projections (Kopanitsa et al., 2006) in well oxygenated (95% O_2_-5% CO_2_), ice-cold sucrose-containing cutting solutions (85 mM NaCl, 75 mM sucrose, 2.5 mM KCl, 25 mM glucose, 1.25 mM NaH_2_PO_4_, 4 mM MgCl_2_, 0.5 mM CaCl_2_, and 24 mM NaHCO_3_). Two slices prepared at intermediate levels of the dorsal-ventral axis of hippocampus from each hemisphere were visually confirmed to have preserved CA3-DG structures, which were then used for experiments. For VSD imaging experiments, slices were first incubated in the cutting solution for 30 min at 32°C, and then transferred for dye staining at room temperature (22°C) for 1 h in oxygenated ACSF (126 mM NaCl, 2.5 mM KCl, 26 mM NaHCO_3_, 2 mM CaCl_2_, 2 mM MgCl_2_, 1.25 mM NaH_2_PO_4_, and 10 mM glucose) containing 0.12 mg/ml of the absorption VSD, NK3630 (Kankoh-Shikiso Kenkyusho), then maintained in the regular ACSF before use. For LSPS experiments, slices were first incubated in the sucrose-containing ACSF for 30 min to 1 h at 32°C and then transferred to the regular ACSF before use. Throughout the cutting, incubation and recording processes, the solutions were continuously supplied with 95% O_2_–5% CO_2_. We used standard open recording chambers which maintained slice health and viability well, as evidenced by the measurement of slice activities for periods lasting >6 h.

### Fast VSD imaging

Our overall system of electrophysiological recordings, photostimulation and imaging was described previously (Xu et al., 2010a). The solution was fed into the slice recording chamber through a pressure-driven flow system with pressurized 95% O_2_–5% CO_2_. The perfusion flow rate is ∼2 ml/min. Photostimulation via glutamate uncaging was used to evoke excitatory activity in the slices. For photostimulation of VSD imaging, stock solution of MNI-caged-L-glutamate (Tocris Bioscience) was added to 20 ml of ACSF for a final concentration of 0.2 mM caged glutamate. Caged glutamate was present in the bath solution, and only turned active through focal UV photolysis. The slice image was acquired by a high resolution digital CCD camera, which in turn was used for guiding and registering photostimulation sites. A laser unit (DPSS Lasers) was used to generate a 355-nm UV laser for glutamate uncaging. Short pulses of laser flashes (1 ms, 20 mW) were controlled using an electro-optical modulator and a mechanical shutter. The laser beam formed uncaging spots, each approximating a Gaussian profile with a width of ∼100 µm laterally at the focal plane.

During VSD imaging experiments, 705-nm light trans-illuminated brain slices and voltage-dependent changes in the light absorbance of the dye were captured by the MiCAM02 fast imaging system (SciMedia USA). The restricted spectral illumination was supplied by optically band filtered white light from an Olympus tungsten-halogen lamp up to 100 W. Optical recording of VSD signals was performed under a 2× or 4× objective with a sampling rate of 4.4 ms per frame [frame resolution 88 (w) × 60 (h) pixels]. VSD imaging of evoked activity was triggered and synchronized with each laser photostimulation at specified cortical sites. For each trial, the VSD imaging duration was 2000 frames including 500 baseline frames with an intertrial interval of 12 s.

VSD signals were originally measured by the percentage change in pixel light intensity [ΔI/I%; the percentage change in the intensity (ΔI) at each pixel relative to the initial intensity (I)]. In addition, the mean and standard deviation (SD) of the baseline activity of each pixel across the 50 frames preceding photostimulation was calculated, and VSD signal amplitudes were then expressed as SD multiples above the mean baseline signal for display and quantification. The activated pixel was empirically defined as the pixel with the amplitude ≥1 SD above the baseline mean of the corresponding pixel’s amplitude (equivalent to the detectable signal level in the original VSD maps of ΔI/I%). VSD images were smoothed by convolution with a Gaussian spatial filter (kernel size: five pixels; SD (σ): one pixel), and a Gaussian temporal filter (kernel size: three frames; δ: one frame). In the present study, single-trial VSD signals were of sufficiently high amplitudes and could be discerned from background noise; no averaging over multiple trials was used for data presentation unless specified. Images were displayed and analyzed using custom-made MATLAB programs. To quantify VSD response strength of photostimulation-evoked neural activities, the average number of activated pixels and average response amplitude within the defined window of analysis were measured for each trial.

### Electrophysiology and LSPS

To perform whole-cell recordings, individual neurons were visualized at high magnification (60× objective), and were patched with a glass electrode of 4–6 MΩ resistance that was filled with an internal solution containing 126 mM K-gluconate, 4 mM KCl, 10 mM HEPES, 4 mM ATP-Mg, 0.3 mM GTP-Na, and 10 mM phosphocreatine; pH 7.2, 300–305 mOsm. The internal solution also contained 0.1% biocytin for cell labeling and morphologic identification. Once a stable whole-cell recording was achieved with a good access resistance (usually <20 MΩ), basic electrophysiological properties were examined through hyperpolarizing and depolarizing current injections. For LSPS experiments, the microscope objective was switched from 60× to 4×. Stock solution of MNI-caged-L-glutamate was added to 20 ml of ACSF for a final concentration of 0.2 mM caged glutamate. 

During mapping experiments, photostimulation was usually applied to 16 × 16 patterned sites (with an intersite space of 100 µm^2^) covering the whole hippocampus in a nonraster, nonrandom sequence to avoid revisiting the vicinity of recently stimulated sites. Whole-cell voltage-clamp recordings were made from the recorded neurons to measure photostimulation-evoked EPSC responses at the holding potential around -70 mV, which was based on the empirically determined GABAergic reversal potentials at the recorded mouse ages. In separate experiments, the recorded neurons were held at +5 mV in the voltage clamp mode with a cesium-containing internal solution to map IPSC responses.

Photostimulation data analysis has been described (Shi et al., 2010). Photostimulation can induce two major forms of excitatory responses (Xu and Callaway, 2009; Shi et al., 2010): (1) direct glutamate uncaging responses (direct activation of the recorded neuron’s glutamate receptors); and (2) synaptically mediated responses (e.g., EPSCs) resulting from the suprathreshold activation of presynaptic excitatory neurons. Responses within a 10-ms window from laser onset are considered as direct uncaging activations. Synaptic currents with such short latencies are not possible because they occur before the generation of action potentials in photostimulated neurons. To exclude the direct responses, candidate EPSCs with their arrival times occurring within the direct response window (within 10 ms of the laser onset) are dismissed. Similarly, for inhibitory postsynaptic responses, we only included actual presynaptic inhibitory input (resulting from somatic firing of inhibitory neurons at stimulated locations) to construct inhibitory input maps. A new technique that combines the design of a bank of approximate matched filters with a detection and estimation theory was implemented for automated detection and extraction of photostimulation-evoked EPSCs or IPSCs (Shi et al., 2010). As for individual map construction, input measurements from different stimulation sites were assigned to their corresponding anatomic locations in the hippocampus; color-coded maps of average input amplitude and the number of events per site were plotted to illustrate overall input pattern to the recorded cell. The input amplitude of each stimulation site was the sum of individual EPSCs or IPSCs from that photostimulation site within the analysis window of 150 ms after photostimulation, with the baseline spontaneous response subtracted from the photostimulation response of the same site. LSPS-evoked synaptic responses were quantified across the 16 × 16 mapping grid for each cell, and two to four individual maps were averaged per recorded cell, reducing the likelihood of incorporating noise events in the analysis window (150 ms). The input measurement represents synaptic charge in the specified analysis window. However, for consistency with previous studies, the values are normalized with the duration of the analysis window and presented as picoamperes (pA). To quantitatively compare input strength and connections across cell groups, we measured the total sum of the ESPCs or IPSCs input strength across the map sites for individual cells, and assessed the extent of synaptic connections by measuring the number of stimulation locations providing synaptic input in the mapping region. We also compared EPSC or IPSC latencies of data maps across the cell groups.

After physiologic assays had been completed, the brain slices were fixed in 4% paraformaldehyde in PBS overnight and transferred to 30% sucrose solution in PBS. The slices were stained against biocytin with 1:1000 Alexa Fluor 488-conjugated streptavidin (Jackson ImmunoResearch) to show the morphology of the recorded cells. Neuron reconstructions were computer-assisted and based on stacks of optical sections acquired by a confocal microscope (LSM 700, Carl Zeiss).

### Statistical analysis

Data were presented as mean ± SE. For statistical comparisons between groups, the data were checked for normality distribution and equal variance. If the criteria were met, a *t* test was performed to compare two groups; when the criteria were not met, a Mann–Whitney *U* test was used. For statistical comparisons across more than two groups, we used the Kruskal–Wallis test (nonparametric one-way ANOVA) and the Mann–Whitney *U* test for group comparisons. One-way ANOVA with Tukey *post hoc* test were used for parametric group comparisons as well. In all experiments, the level of statistical significance was defined as *p* < 0.05.

## Results

### Projection-based viral targeting of hilar mossy cells for circuit mapping

**Figure 1.**
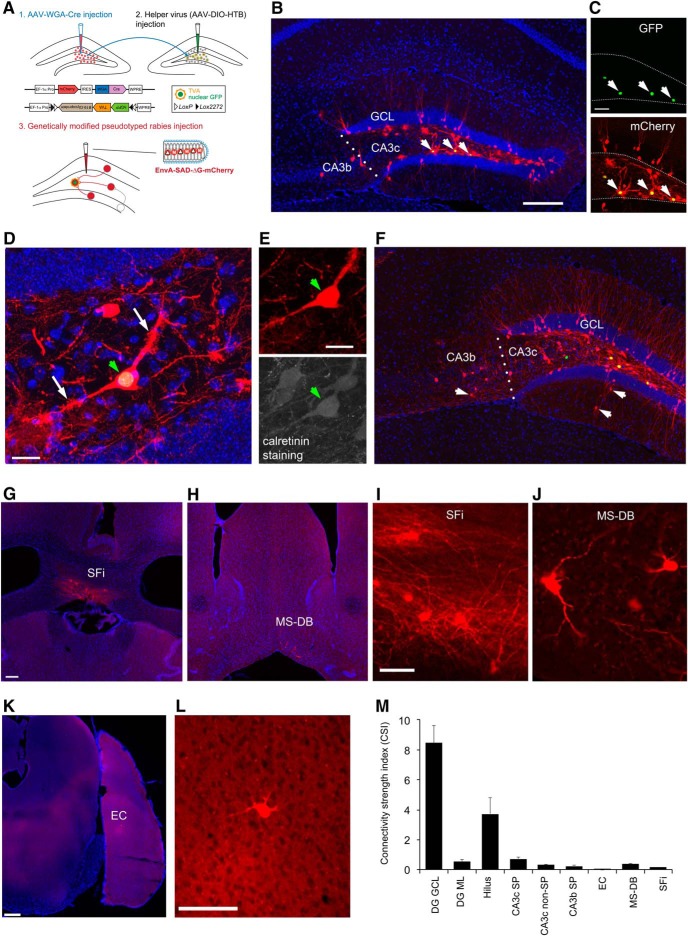
Viral genetic targeting of hilar mossy cells for rabies-based mapping of circuit connections *in vivo***. *A***, The schematic illustrates our projection-based mossy cell targeting strategy (Gradinaru et al., 2010). The WGA-Cre AAV was unilaterally injected into one DG (1) in the wild-type C57BL/6 mouse to express Cre in some mossy cells in the contralateral DG, while the Cre-dependent helper AAV for rabies tracing was injected into the contralateral DG of the same mouse (2). The construct design is shown for WGA-Cre (top) and Cre-dependent (bottom) AAV vectors. At three weeks following the AAV injection, the genetically modified rabies was injected (3) for monosynaptic tracing of direct inputs to the targeted mossy cells. ***B***, Example starter mossy cells (expressing both nuclear EGFP and mCherry) and their presynaptic neurons (only labeled by rabies mCherry expression) in the DG and CA3 at the helper AAV and rabies injection site. The arrows point to starter neurons. The sale bar (200 μm) in ***B*** applies to both ***B*** and ***F***. ***C***, The starter neurons have GFP expression (top) from the helper AAV genome and mCherry expression (bottom) from the rabies genome. Scale bar, 50 μm. ***D***, The starter neurons show mossy appearances that defines hilar mossy cells. The white arrows point to numerous thorny excrescences at the cell’s proximal dendrites. Scale bar, 25 μm. ***E***, The starter cell pointed by the green arrowhead is also immunopositive for CR staining. Scale bar, 25 μm. ***F***, Another example showing starter mossy cells and their presynaptic neurons in the DG and CA3 (with some labeled cells in CA3b). The arrows point to putative inhibitory neurons labeled in both DG molecular layer and CA3 nonpyramidal layer. ***G***, ***I***, rabies-labeled neurons in SFi. The scale bar in ***G*** (200 μm) applies to both ***G*** and ***H***; the scale bar in ***I*** (25 μm) applies to both ***I*** and ***J***. ***H***, ***J***, Rabies-labeled neurons in the MS-DB. ***K***, ***L***, Only one labeled entorhinal neuron identified in the example case. Scale bars, 200 μm (***K***) and 50 μm (***L***). ***M***, The graph shows quantification of average strengths of specific circuit connections to targeted mossy cells. The input CSI is defined as the ratio of the number of labeled presynaptic neurons in a specified structure versus the number of starter mossy neurons. Data are presented as mean ± SE. See Table 1 for more information.

It is known that the monosynaptic interhemispheric projection arises from the hilar mossy cells within the dentate hilus (Freund and Buzsáki, 1996; Ratzliff et al., 2004). The projection-based viral strategy was first established for optogenetic control of hilar mossy cells (Gradinaru et al., 2010), by taking advantage of their axonal projections to the contralateral DG. In this study, we are able to selectively target hilar mossy cells for circuit connection mapping. As illustrated in Figure 1*A*, WGA-Cre AAV was unilaterally injected into the DG in one hemisphere in the wild-type C57BL/6 mouse to express Cre-recombinase in mossy cells in the DG in the other hemisphere. Then the Cre-dependent rabies tracing system (Sun et al., 2014), including the helper AAV and genetically modified, pseudotyped rabies was applied to map circuit connections to Cre targeted mossy cells in the intact brain (Fig. 1*A*). To map direct synaptic connections to selected neurons, this approach requires that a starter population expresses both the EnvA receptor (TVA) and rabies glycoprotein (RG) via the helper AAV. This population is then selectively infected with an EnvA-pseudotyped, RG-deleted rabies virus (EnvA-SADΔG-mCherry rabies). Together, this conditional intersection results in transcomplementation and monosynaptic retrograde spread of the rabies virus to presynaptic neurons of the starter population. The AAV-targeted subset of mossy cells are identified by their nuclear GFP expression from the helper AAV genome (Fig. 1*B*,*C*). Starter mossy cells in brain sections are unambiguously identified by their GFP and mCherry expression from the helper AAV and ΔG-mCherry rabies genomes, respectively. Because rabies replicates its core within starter neurons and their presynaptic neurons, this produces intense fluorescence with strong mCherry expression and thus reveals detailed cellular structures (Fig. 1*D*,*E*).

**Figure 2.**
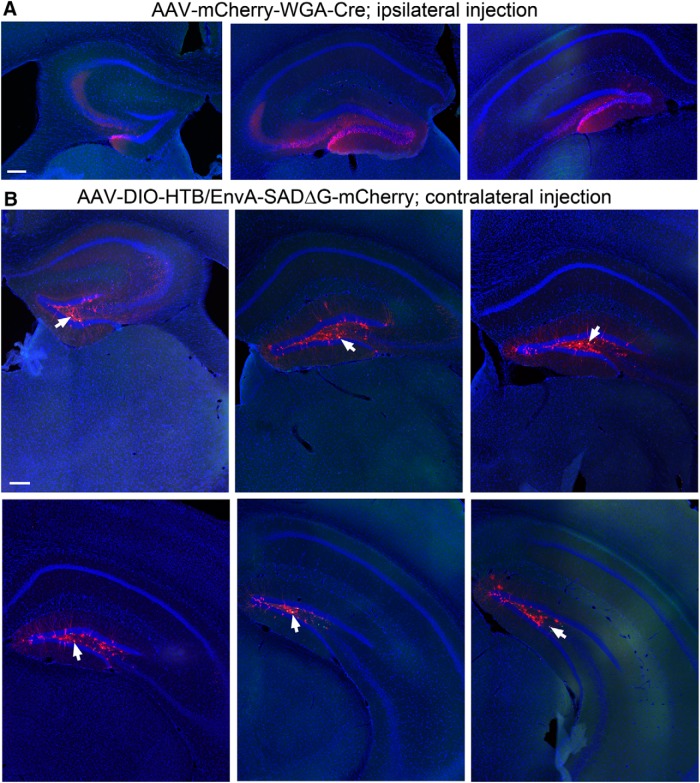
Example section images showing the ipsilateral DG with the WGA-Cre AAV injection, and spatially restricted starter neurons in the dentate hilus in the contralateral DG. ***A***, The AAV-mCherry-IRES-WGA-Cre strongly labels the cells (mCherry fluorescence, red) in the granule cell layer and the hilus, delineated by DAPI fluorescence (blue) in coronal sections at the septal, intermediate, and temporal levels. Scale bar, 200 μm. ***B***, WGA-Cre activated helper AAV expression (nuclear EGFP fluorescence) is confined in the contralateral hilus of six different coronal sections at the septal to temporal levels. Arrows point to the helper AAV and rabies double-labeled starter neurons in the hilus. Scale bar, 200 μm.

**Figure 3.**
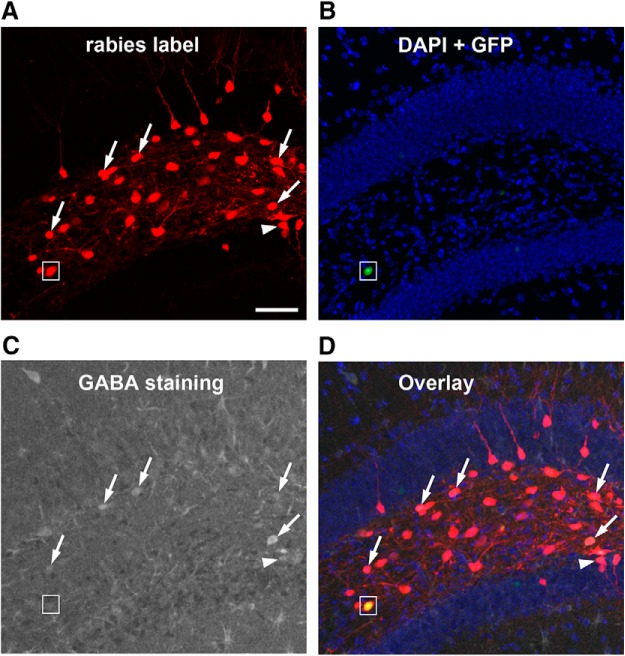
GABA immunostaining of rabies-labeled neurons indicate that mossy cells receive inputs from both GABAergic and non-GABAergic neurons in local circuits. ***A***, Rabies virus-labeled neurons (mCherry expression only, red) in the DG are direct presynaptic neurons of targeted hilar mossy cells. ***B***, DAPI staining (blue) shows the anatomic structure of the DG, and nuclear EGFP label (small white box) shows a mossy starter neuron with AAV helper virus infection. ***C***, GABA immunostaining (gray) shows GABAergic cells. ***D***, The overlay image shows rabies-labeled hilar cells that are immunopositive for GABA (indicated by the white arrows). The white arrowhead points to a rabies-labeled neuron that is GABA immunopositive from the dentate granule cell layer. The small white box shows the hilar mossy starter neuron that lacks GABA staining. Scale bar, 200 μm.

Through carefully calibrated injections of the WGA-Cre AAV in one side of the DG (Fig. 2*A*) and spatially restricted injection of the Cre dependent helper AAV (labeling starter mossy cells) in the other side of the DG (Fig. 2*B*), we were able to achieve 4 excellent cases for targeted mossy cell tracing from 15 animals. As per our exclusion criteria, cases showing potential leaks to CA3 were excluded for analysis. In the accepted four cases that unambiguously passed this criteria, we were able to label a small number of starter hilar mossy cells (36-70 cells; see Table 1) distributed along the dorsal-ventral axis of DG (Fig. 2*B*). We have confirmed the selectivity of mossy cell targeting, based on morphologic and immunocytochemical criteria established in previously published studies (Scharfman, 2007). The location and shapes of the mossy cell somata and their characteristic large and thick dendrites in the hilus robustly distinguishes these cells from other types of hilar neurons, dentate granule cells or CA3 pyramidal cells (Fig. 1*B*–*F*). Consistently, the nuclear EGFP-tagged, mCherry-filled mossy cells (“starter neurons”) have mossy-like thorny excrescences on their proximal dendrites. In addition, immunostaining of selected sections at more ventral levels along the dorsal-ventral axis of DG show that these cells are CR expressing but GABA negative, which is known neurochemically for mouse mossy cells, thus fulfilling additional strict criteria (Figs. 1*D*,*E* and 3).

**Table 1.** Quantification of rabies-labeled presynaptic inputs to hilar mossy cells

A. Individual case summary										
	Starter neurons	DG GCL	DG ML	DG Hilus	CA3c SP	CA3c non-SP	CA3b SP	EC	MS-DB	SFi
Case 1561 (total 365 neuron counts)										
Number of neurons	36	227	15	59	16	12	5	0	17	10
CSI		6.31	0.42	1.64	0.44	0.33	0.14	0.00	0.47	0.28
% of total labeling		62.19	4.11	16.16	4.38	3.29	1.37	0.00	4.66	2.74
Case 1562 (total 431 neuron counts)										
Number of neurons	39	262	15	79	22	15	4	1	12	11
CSI		6.72	0.38	2.03	0.56	0.38	0.10	0.03	0.31	0.28
% of total labeling		60.79	3.48	18.33	5.10	3.48	0.93	0.23	2.78	2.55
Case 2543 (total 1247 neuron counts)										
Number of neurons	70	660	33	366	78	20	21	0	20	3
CSI		9.43	0.47	5.23	1.11	0.29	0.30	0.00	0.29	0.04
% of total labeling		52.93	2.65	29.35	6.26	1.60	1.68	0.00	1.60	0.24
Case 2544 (total 1333 neuron counts)										
Number of neurons	62	702	59	369	49	22	27	2	31	6
CSI		11.32	0.95	5.95	0.79	0.35	0.44	0.03	0.50	0.10
% of total labeling		52.66	4.43	27.68	3.68	1.65	2.03	0.15	2.33	0.45

B. Summary data of all 4 cases									
	DG GCL	DG ML	DG Hilus	CA3c SP	CA3c non-SP	CA3b SP	EC	MS-DB	SFi
CSI	8.44	0.56	3.71	0.73	0.34	0.24	0.01	0.39	0.17
SE	1.18	0.13	1.10	0.15	0.02	0.08	0.01	0.06	0.06
% of total labeling	57.14	3.67	22.88	4.85	2.51	1.50	0.10	2.84	1.50
SE	2.53	0.39	3.30	0.55	0.51	0.23	0.06	0.65	0.67

As every one in three sections across the whole brain series was used for mapping rabies-labeled presynaptic neurons of targeted dentate granule cells, the actual total number of labeled neurons in each case would be estimated three times of the total neuron counts listed in the table. The labeled neurons are predominantly distributed in the ipsilateral hemisphere. The neuronal count for each structure in each case is pooled from both hemispheres if there are any labeled neurons in the contralateral hemisphere. The percentage of total labeled neurons is calculated as the percentage of the number of labeled presynaptic neurons in a specified structure versus the overall total presynaptic neuron count in each case. The input CSI is defined as the ratio of the number of labeled presynaptic neurons in a specified structure versus the number of starter neurons.

DG GCL, DG granule cell layer; DG ML, DG molecular layer; CA3b-c SP, CA3b-c pyramidal cell layer; CA3c non-SP, CA3c nonpyramidal cell layer.

We follow the basic nomenclature of Lorente de Nó (1934) and Ishizuka et al. (1990) to describe hippocampal sub-regions. The terms of proximal (nearer the DG) and distal (farther from the DG) are used to designate positions along the transverse axis of CA3 (Ishizuka et al., 1990; Fig. 1). The midline of the fimbria separates CA3b and CA3a. Excitatory neurons (dentate granule cells, CA3 pyramidal cells) and inhibitory interneurons are identified based on their laminar locations and morphology, and further confirmed by GABA immunostaining in selected sections. Quantitative strengths of specific circuit connections to mossy cells are assessed, and the input connection strength index (CSI) is defined as the ratio of the number of labeled presynaptic neurons in a specified structure versus the number of starter neurons. In addition, the percentage of total labeled neurons for a specified structure is calculated as the percentage of the number of labeled presynaptic neurons in a specified structure versus the overall total presynaptic neuron count in each case (Tables 1 and 2).

**Table 2.** Quantification of rabies-labeled presynaptic inputs to dentate granule cells

	**Starter neuron**	**DG GCL**	**DG ML**	**DG Hilus**	**CA1 non-SP**	**CA3a SP**	**CA3a non-SP**	**CA3b SP**	**CA3b non-SP**	**CA3c SP**	**CA3c non-SP**	**PRh**	**LEnt**	**mEnt**	**MS-DB**	**Subicu-lum**	**SuM**	**Raphe**
A. Individual case summary																		
**Case 1711 (a total of 2421 neuron counts)**																		
Number **of neurons**	128	23	10	261	13	125	24	80	24	33	7	40	1285	286	182	8	15	1
**CSI**		0.18	0.08	2.04	0.10	0.98	0.19	0.63	0.19	0.26	0.05	0.31	10.04	2.23	1.42	0.06	0.12	0.01
**% of total labeling**		0.95	0.41	10.78	0.54	5.16	0.99	3.30	0.99	1.36	0.29	1.65	53.08	11.81	7.52	0.33	0.62	0.04
**Case 1713 (a total of 1810 neuron counts)**																		
Number **of neurons**	119	18	7	168	18	48	8	34	24	12	9	73	1048	165	161	8	7	2
**CSI**		0.15	0.06	1.41	0.13	0.40	0.07	0.29	0.20	0.10	0.08	0.61	8.81	1.39	1.35	0.07	0.06	0.02
**% of total labeling**		0.99	0.39	9.28	1	2.65	0.44	1.88	1.33	0.66	0.50	4.03	57.90	9.12	8.90	0.44	0.39	0.11
**Case 1868 (a total of 3192 neuron counts)**																		
Number **of neurons**	228	35	16	421	29	147	29	96	35	53	17	77	1669	315	196	10	35	5
**CSI**		0.15	0.07	1.85	0.13	0.64	0.13	0.42	0.15	0.23	0.07	0.34	7.32	1.38	0.86	0.04	0.15	0.02
**% of total labeling**		1.10	0.50	13.19	0.91	4.61	0.91	3.01	1.10	1.66	0.53	2.41	52.29	9.87	6.14	0.31	1.10	0.16
**Case 1867 (a total of 1441 neuron counts)**																		
Number **of neurons**	88	15	11	137	28	63	29	39	24	32	6	42	693	198	106	4	7	0
**CSI**		0.17	0.13	1.56	0.30	0.72	0.33	0.44	0.27	0.36	0.07	0.48	7.88	2.25	1.20	0.05	0.08	0.00
**% of total labeling**		1.04	0.76	9.51	1.94	4.37	2.01	2.71	1.67	2.22	0.42	2.91	48.09	13.74	7.36	0.28	0.49	0.00
**Case 1864 (a total of 509 neuron counts)**																		
Number **of neurons**	32	5	7	45	8	17	5	10	4	7	2	9	269	66	52	1	2	0
**CSI**		0.16	0.22	1.41	0.25	0.53	0.16	0.31	0.13	0.22	0.06	0.28	8.41	2.06	1.63	0.03	0.06	0.00
**% of total labeling**		0.98	1.38	8.84	1.57	3.34	0.98	1.96	0.79	1.38	0.39	1.77	52.85	12.97	10.22	0.20	0.39	0.00

B. Summary data of all 5 cases																	
	**DG GCL**	**DG ML**	**DG Hilus**	**CA1 non-SP**	**CA3a SP**	**CA3a non-SP**	**CA3b SP**	**CA3b non-SP**	**CA3c SP**	**CA3c non-SP**	**PRh**	**LEnt**	**mEnt**	**MS-DB**	**Subicu-lum**	**SuM**	**Raphe**
**CSI (Mean)**	0.16	0.11	1.65	0.18	0.65	0.17	0.42	0.19	0.23	0.07	0.40	8.49	1.86	1.29	0.05	0.09	0.01
**SE**	0.01	0.03	0.13	0.04	0.10	0.04	0.06	0.03	0.04	0.00	0.06	0.46	0.20	0.13	0.01	0.02	0.00
**% of total labeling**	1.01	0.69	10.32	1.19	4.03	1.07	2.57	1.17	1.46	0.43	2.56	52.84	11.50	8.03	0.31	0.60	0.06
**SE**	0.03	0.18	0.79	0.25	0.45	0.26	0.28	0.15	0.25	0.04	0.43	1.56	0.88	0.70	0.04	0.13	0.03

As every one in three sections across the whole brain series were used for mapping rabies-labeled presynaptic neurons of targeted dentate granule cells, the actual total number of labeled neurons in each case would be estimated three times of the total neuron counts listed in the table. The labeled neurons are predominantly distributed in the ipsilateral hemisphere. The neuronal count for each structure in each case is pooled from both hemispheres if there are any labeled neurons in the contralateral hemisphere. The percentage of total labeled neurons is calculated as the percentage of the number of labeled presynaptic neurons in a specified structure versus the overall total presynaptic neuron count in each case. The input CSI is defined as the ratio of the number of labeled presynaptic neurons in a specified structure versus the number of starter neurons.

DG GCL, DG granule cell layer; DG ML, DG molecular layer; CA1 non-SP, CA1 nonpyramidal cell layers; CA3a-c SP, CA3a-c pyramidal cell layer; CA3a-c non-SP, CA3a-c nonpyramidal cell layer; LEnt, lateral EC; mEnt, medial EC; SuM, supramammillary nucleus; Raphe, raphe nuclei.

The mossy cells receive predominant intrahippocampal inputs, consisting of both excitatory and inhibitory inputs from within the DG and CA3. Supported by earlier slice recording work (Jackson and Scharfman, 1996), our *in vivo* circuit mapping finds that overall dentate granule cell input to hilar mossy cells is the strongest, with the labeled granule cells accounting for ∼57% of the total presynaptic input neurons to mossy cells. The average CSI of the granule cells is 8.44 ± 1.18 (mean ± SE; Fig. 1*B*,*F*,*M*; Table 1). Our study also reveals extensive labeling of hilar neurons with mossy cell-like appearance. The overall labeled hilar neurons (with both GABA+ and GABA- cell types; Fig. 3) make up ∼23% of the total labeled neurons (CSI: 3.71 ± 1.1; Table 1). Approximately 2/3 of the rabies-labeled hilar cells (132 out of 208 neuron counts pooled from three cases) are GABA- in selected sections. This indicates that excitatory mossy cells are interconnected locally through chemical synapses, considering that rabies virus does not cross gap junctions (Ugolini, 2011). Excitatory neurons in the CA3 pyramidal cell layer provide a moderate degree of excitatory inputs to mossy cells. The rabies-labeled CA3 cells are not only from proximal (CA3c) but also from the CA3 mid-subfield (CA3b; Fig. 1*B*,*F*), accounting for ∼4.9% and 1.5% of the total presynaptic input neurons to mossy cells, respectively. Their average CSIs are 0.73 ± 0.15 and 0.24 ± 0.08 (*p* < 0.05; Fig. 1*M*). In contrast, the entorhinal input is very minor, only one or two cells labeled in entorhinal cortex (EC) in two out of the four mossy cell-tracing cases (Fig. 1*K*,*L*).

Compared with excitatory inputs, our prior understanding of the inhibitory input sources to mossy cells was less clear. Here, we show that the mossy cells receive inhibitory inputs parallel to excitatory inputs from within the DG and CA3. The inhibitory neurons (identified by GABA staining in some cases and stereotyped localization in all cases) in the DG molecular layer and CA3 nonpyramidal cell layers make up ∼3.7% and 2.5% of the total presynaptic input neurons to mossy cells, respectively (Fig. 1*F*). Their respective average CSIs are 0.56 ± 0.13 and 0.34 ± 0.02. Local hilar inhibitory neurons are also an important source of inhibitory input to mossy cells, as GABA immunostaining indicates that ∼38% of rabies-labeled hilar neurons (Fig. 3) are inhibitory neurons. This is in agreement with the electrophysiological slice recording study reporting that hilar interneurons show synapse specificity and preferentially inhibit mossy cells (Larimer and Strowbridge, 2008). In addition, we also observed ∼22% of the rabies-labeled neurons in the dentate granule cell layer are immunopositive for GABA (Fig. 3), which indicate mossy cells also receive local inhibitory inputs from interneurons in the granule cell layer.

**Figure 4.**
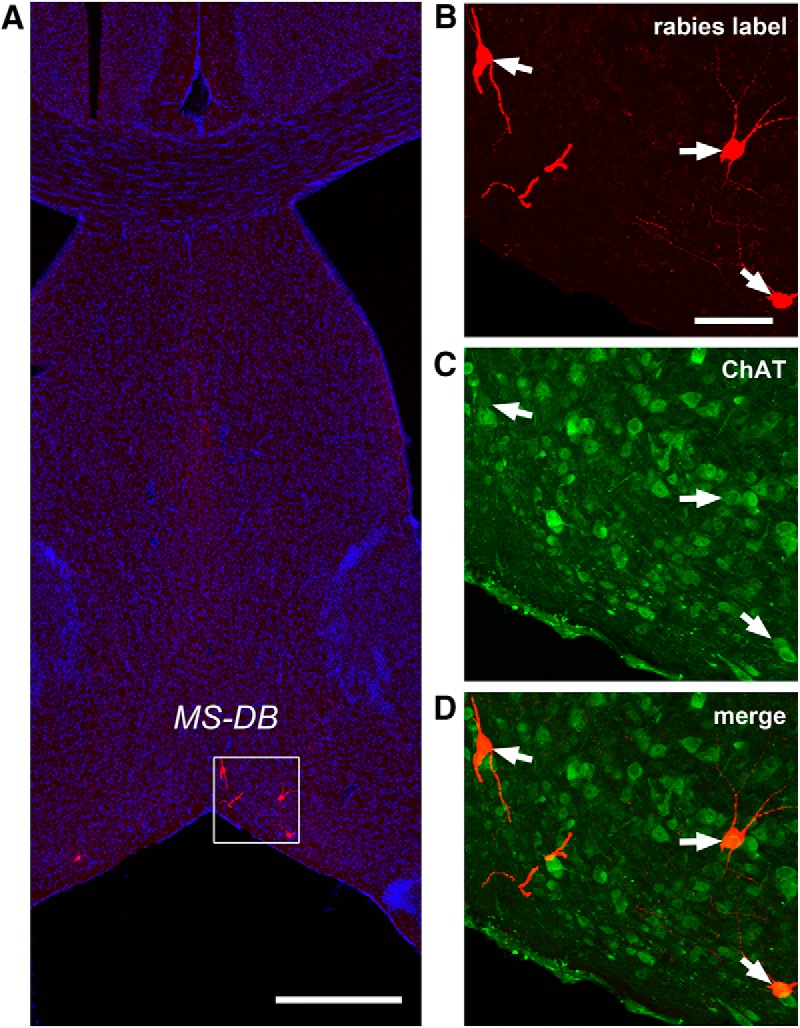
Cholinergic neurons in the MS-DB provide direct synaptic inputs to hilar mossy cells. Rabies virus-labeled neurons in the MS-DB are consistently immunopositive for ChAT but not GABA. ***A***, An example MS-DB slice shows rabies-labeled neurons (red) and DAPI staining (blue). Scale bar, 500 μm. ***B–D***, Enlarged views of the white box region shown in ***A*** with ChAT immunostaining (green). The arrows point to rabies-labeled neurons that are immunopositive for ChAT. Scale bar, 200 μm.

While we cannot expect our rabies method to label every input to each neuron (Callaway and Luo, 2015), rabies tracing appears to work in an effective fashion as established in our previous work (Sun et al., 2014). Because the rabies virus labels inputs to different cell types in a similar manner (Ugolini, 2011), we can assess the relative number of inputs from each source to each target cell type and quantify the number of cells that are labeled at various input locations following rabies tracing of different types of postsynaptic cells in the DG. In contrast to single-cell targeting (Marshel et al., 2010; Rancz et al., 2011), this approach benefits from targeting a small population of Cre+ postsynaptic cells and can provide weighted connection strengths for defined cell types. Because the number of postsynaptic starter cells and the number of direct presynaptic labeled cells in specified structures across the entire brain can be quantitatively determined, this approach allows for assessment of the relative abundance of connected populations using the CSI measurements. In this study we find that long range inputs to mossy cells are numerically sparse, and that they are only identified readily from the MS-DB and the septofimbrial nucleus (SFi; Fig. 1*G*–*J*). The septal inputs are composed of ∼2.8% of the total labeled presynaptic input neurons, with a CSI of 0.39 ± 0.06. Approximately 75% of the rabies-labeled MS-DB neurons are cholinergic, revealed by robust ChAT immunoreactivity in rabies-labeled cells, while they essentially show no GABA immunostaining, thus excluding a significant GABAergic contribution from those basal forebrain nuclei (Fig. 4). The SFi has been reported to have neurons that predominantly use ATP as a fast neurotransmitter with a likely cotransmitter of glutamate (Edwards et al., 1992; Sperlágh et al., 1998) and project to medial habenula. The SFi input to mossy cells is reported for the first time here; the labeled neurons account for 1.5% of the total labeled presynaptic input neurons, with a CSI of 0.17 ± 0.06 (Table 1).

### Circuit connections to dentate granule cells

To further understand circuit connections between hilar mossy cells and dentate granule cells, the Cre-dependent rabies tracing system was applied to map direct synaptic connections to dentate granule cells using D1-Cre transgenic mice, in which the dentate granule cell layer expresses the Cre recombinase (Lemberger et al., 2007). The targeted granule cells are located in hippocampal sections at a more ventral level, matching the position with a large portion of mossy starter neurons (Fig. 2*A*,*B*). The starter granule cells ranged from 32-228 cells for the five cases analyzed.

**Figure 5.**
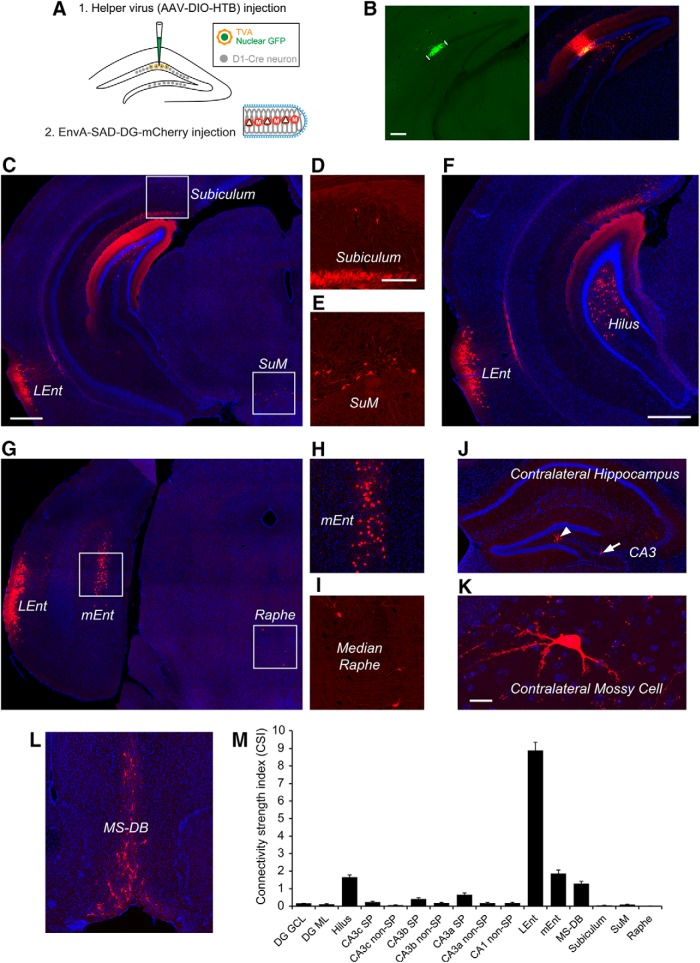
Cre-dependent rabies tracing of circuit connections to dentate granule cells. ***A***, The schematic illustrates the use of Cre dependent helper AAV and a D1-Cre transgenic mouse, in which the dentate granule cell layer expresses Cre recombinase (Lemberger et al., 2007) for Cre-dependent monosynaptic rabies tracing. ***B***, Spatially restricted iontophoretic injection of the helper AAV (AAV-DIO-HTB) in the temporal DG of the D1-Cre mouse (left), followed by EnvA-SADΔG-mCherry rabies infection (right). The short white lines indicate restricted GFP-expressing helper AAV infection in the granule cell layer. Scale bar, 200 μm. ***C–L***, Dentate granule cells receive very strong input from lateral and medial EC (***C***, ***F*–*H***), extensive input from mossy cells at the temporal hilus (***F***), and inputs from medial septum (MS-DB) (***L***). Other long range inputs come from the subiculum, supramammillary nucleus (SuM), median raphe nucleus (***D***, ***E***, ***I***) as well as contralateral mossy cells (***J*** and ***K***). Scale bars in ***C*** (500 μm) applies to ***G***, ***J***, ***L***; in ***D*** (200 μm) applies to ***E***, ***H***, ***I***; in ***F***, 500 μm; in ***K***, 20 μm. ***M***, The graph shows quantitative strengths of specific circuit connections to targeted dentate granule cells. The input CSI is defined as the ratio of the number of labeled presynaptic neurons in a specified structure versus the number of starter dentate granule neurons. Data are presented as mean ± SE. See Table 2 for more information.

In contrast to principal input sources of mossy cells, dentate granule cells receive a great majority of their inputs from the EC including both lateral and medial EC (Fig. 5*C*,*F*–*H*; Table 2). The lateral EC has the most rabies-labeled neurons with a CSI of 8.49 ± 0.46, which makes up of 53% of the total presynaptic input neurons to the targeted granule cells. For the second most labeled structure, the medial EC comprises 11.5% of the total presynaptic input neurons measured with a CSI of 1.86 ± 0.2. The bulk of EC inputs are reflected by mCherry expressing EC axons that heavily innervate the outer DG molecular layer (Fig. 5*C*,*F*). The dentate granule cells also have a 2.6% of total input from the perirhinal cortex (PRh; CSI: 0.4 ± 0.06), which is located adjacent to the lateral EC (Fig. 5*F*,*G*). The labeled neurons in lateral and medial EC and PRh cortical regions exhibit excitatory neuron morphology, indicating cortical excitatory projections to the granule cells.

**Figure 6.**
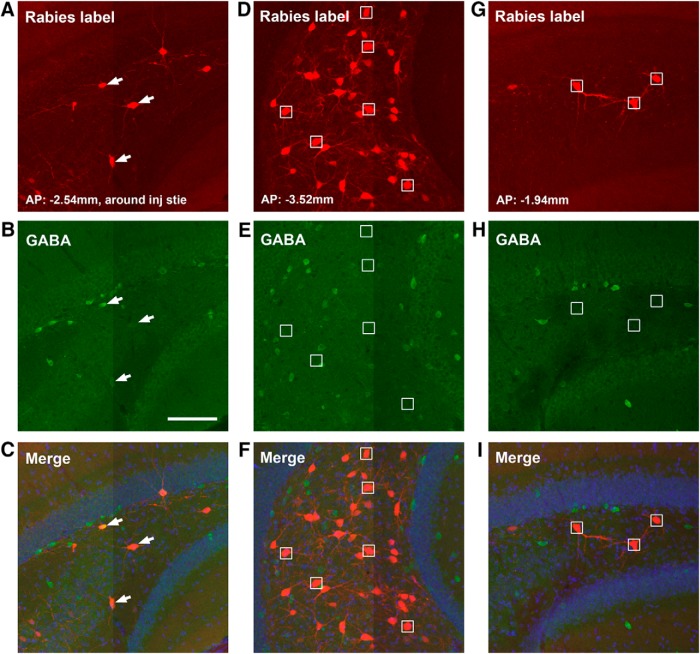
Dentate granule cells tend to receive inputs from GABAergic hilar neurons around the tracer injection site and non-GABAergic hilar mossy neurons from more distal sites. ***A–C***, Example images of GABA immunostaining of rabies-labeled hilar neurons around the injection site of a D1-Cre tracing case. White arrows point to GABA-immunopositive rabies-labeled neurons. ***D–F***, GABA immunostaining of hilar rabies-labeled neurons from a more temporal position to the injection site. There is no colocalization between rabies-labeled neurons and GABA immunostaining. Small white boxes indicate representative non-GABA+ rabies-labeled neurons. ***G–I***, GABA immunostaining of hilar rabies-labeled neurons from a more septal position to the injection site. There is no colocalization between rabies-labeled neurons and GABA immunostaining. Scale bar in ***B*** (100 μm) applies to all the panels. AP numbers indicate the positions of the coronal sections relative to the bregma landmark.

While dentate granule cells provide strong innervation of hilar mossy cells, conspicuously the granule cells receive a very little local input from other granule cells (0.7% of their total input neurons, CSI: 0.11 ± 0.03). In comparison, dentate granule cells receive significant innervation from the hilus (Fig. 5*C*,*F*), as the rabies-labeled hilar cells account for ∼10% of the total presynaptic input neurons (CSI: 1.65 ± 0.13). The granule cells appear to receive strong inputs from hilar mossy cells in both ipsilateral and contralateral hemispheres (Fig. 5*J*,*K*). The hilar mossy cells provide their inputs to granule cells from sections that are both dorsal and ventral to the injection site (Figs. 5*F* and 6*D–I*). In comparison, labeled hilar inhibitory neurons appear more concentrated around the same section plane of the injection site, accounting for 40.3% of total labeled hilar cells around that plane (Fig. 6*A–C*). Overall, 8% of rabies-labeled hilar cells (from 3 different cases) are inhibitory neurons as confirmed by GABA staining.

**Figure 7.**
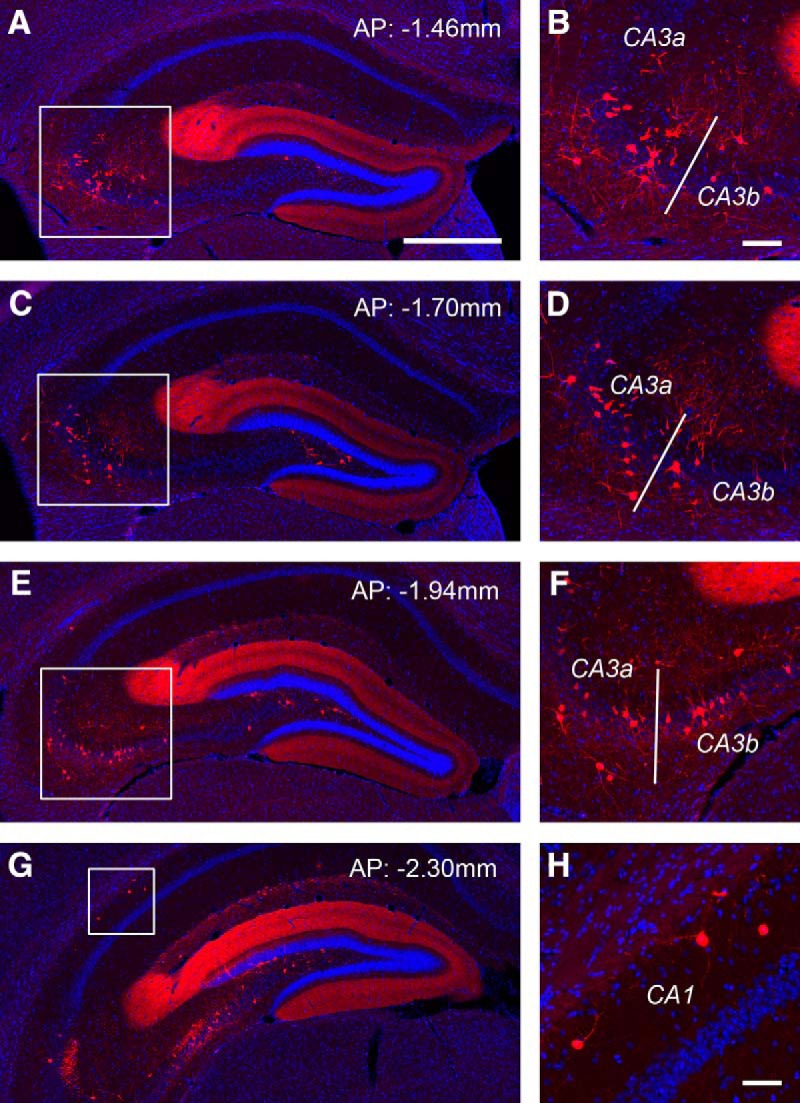
Monosynaptic rabies tracing reveals back projections from CA3 and CA1 to dentate granule cells. ***A–F***, The targeted dentate granule cells receive extensive noncanonical inputs from CA3 in the septal directed hippocampus. AP numbers indicate the positions of the coronal sections relative to the bregma landmark. The example shows that putative CA3a/b excitatory neurons in the pyramidal cell layer and inhibitory neurons outside the pyramidal cell layer provide extensive inputs to dentate granule cells. The fimbria (indicate by the white bar) divides the distal CA3 (CA3a) and the middle CA3 subfield (CA3b). Panels on the right show enlarged views of the white box regions in the left panels. Scale bars, in ***A*** (500 μm) applies to ***A***, ***C***, ***E***, ***G***.; in ***B*** (100 μm) applies to ***B***, ***D***, ***F***. ***G***, ***H***, Putative CA1 inhibitory neurons are also found projecting to dentate granule cells. Scale bar in ***H***, 50 μm.

While anatomic evidence indicates that the axons of some CA3 pyramidal neurons and inhibitory interneurons project back to the DG (Li et al., 1994), the extent of direct back projections from CA3 neurons to dentate granule cells has not been established. Surprisingly, our monosynaptic rabies tracing reveals unexpectedly extensive and direct inputs from CA3 to the dentate granule cells. The noncanonical CA3 inputs (in the context of feedforward DG-CA3 projections) are from all CA3 segments: proximal (CA3c), middle (CA3b), and distal CA3 (CA3a) subfields (Fig. 7). Overall, most of presynaptic CA3 neurons, both excitatory and inhibitory, are located in more dorsal sections relative to targeted DG granule cells (Fig. 7*A*,*C*,*D*,*G*). The rabies-labeled putative excitatory neurons in the pyramidal cell layer account for ∼4%, 2.6%, and 1.5% of the total presynaptic input neurons for CA3a, CA3b, and CA3c, respectively. This may reflect an excitatory back-projection topography with stronger distal CA3 and weaker proximal CA3 projections to DG granule cells (*p* < 0.01; Fig. 5*M*). Thus, the organization of CA3-granule cell back projections is different from that of CA3-mossy cell projections, as proximal CA3 project strongly to mossy cells (Fig. 1*M*). The inputs from putative inhibitory neurons in the nonpyramidal cell layers of CA3 account for ∼1%, 1.2%, and 0.4% of the total presynaptic input neurons to DG for CA3a, CA3b, and CA3c, respectively. The pooled CSIs of CA3a-c for CA3 putative excitatory neurons and inhibitory neurons are ∼1.3 and 0.43, respectively. In addition, dentate granule cells receive weak inputs from CA1 inhibitory neurons (CSI: 0.18 ± 0.04; Fig. 7*G*,*H*), as well as a minor input from the subiculum (CSI: 0.05 ± 0.01; Fig. 5*D*).

**Figure 8.**
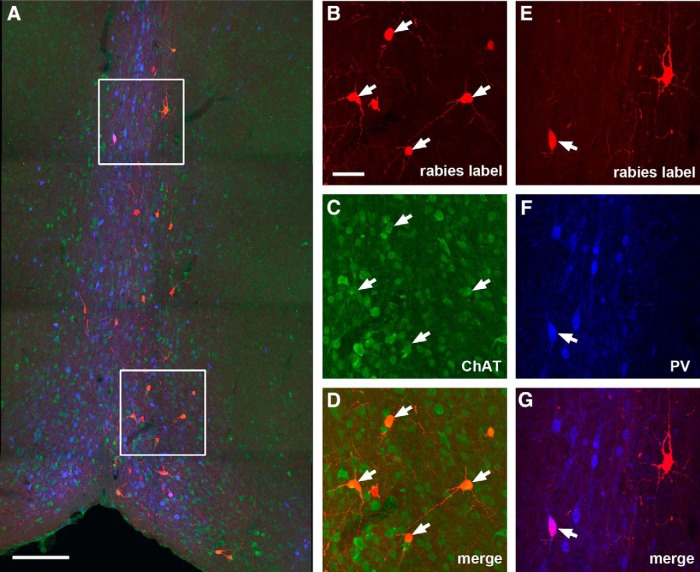
Cholinergic and GABAergic MS-DB inputs to dentate granule cells. ***A***, An example MS-DB section image from a D1-Cre rabies tracing case. The red shows rabies-labeled neurons that are presynaptic to dentate granule cells, the green shows ChAT immunostaining, while the blue shows PV immunostaining. Scale bar, 200 μm. ***B–D***, Enlarged view of the white box region at the top of ***A*** with arrows pointing to ChAT+ rabies-labeled septal neurons. ***E–G***, Enlarged view of the white box region at the bottom of ***A*** with arrows pointing to a PV+ rabies-labeled septal neuron. The scale bar in ***B*** (50 μm) applies to ***B–G***.

Compared with hilar mossy cells, the dentate granule cells receive stronger inputs from the MS-DB (Fig. 5*L*), with a CSI of 1.29 ± 0.13 (*p* < 0.02). Approximately 69% of the rabies-labeled MS-DB neurons (96 out of 138 neuron counts pooled from three cases) are cholinergic; while only ∼5% of them are GABAergic (Fig. 8). Although the granule cells do not receive inputs from the SFi, they receive other minor inputs from the supramammillary nucleus (CSI: 0.09 ± 0.02; Fig. 5*C*,*E*) and the raphe nucleus (CSI: 0.01; Fig. 5*G*,*I*).

### Functional circuit mapping of inputs to mossy cells and dentate granule cells

**Figure 9.**
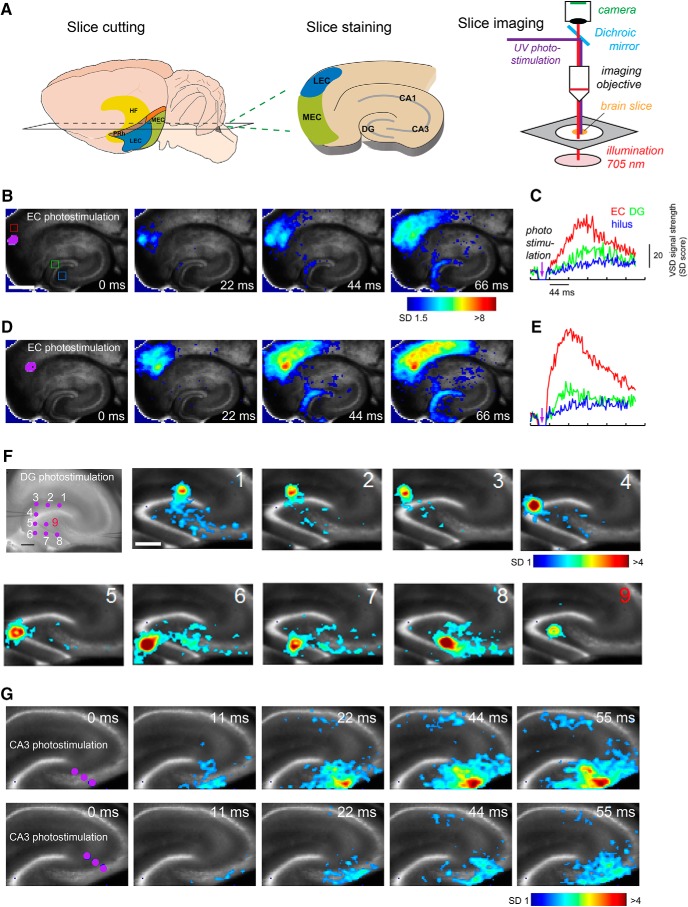
Functional circuit mapping with fast VSD imaging of neural activity supports anatomic rabies tracing results. ***A***, Schematic of living brain slice preparation and the VSD imaging set up. ***B–E***, Spatially restricted photostimulation in EC evokes wide-spread activation in DG (full extent), but weak hilar neuronal activation. ***B***, ***D***, Time series data from VSD imaging of photostimulation-evoked neural activation in the hippocampal formation circuitry. The purple dots (laser stimulation artifact) indicates the photostimulation site. Color-coded activity is superimposed on the background slice image. The color scale codes VSD signal amplitude expressed as SD multiples above the mean baseline. The stronger activation is indicated by the warmer color. Scale bar, 500 μm. ***C***, ***E***, Time course plots of VSD signal from the regions of interest (EC, DG granule cell layer, hilus) indicated by the colored rectangles (red, green, and blue) in the first image frame in ***B***, respectively, starting from the baseline of 22 ms preceding the photostimulation onset. No baseline drift was corrected. ***F***, Spatially restricted photostimulation of dentate granule cells activate hilar and CA3 responses, but hilar photostimulation does not cause significant DG activation. The imaging of individual stimulation at nine different sites (including one hilus stimulation, number 9) was performed; the peak response image frames are plotted at 44 ms after photostimulation. Scale bars: 250 μm (first panel) and 250 μm (second panel), for both ***F*** and ***G***. ***G***, Time series data from VSD imaging of photostimulation-evoked neural activation in proximal CA3b with simultaneous stimulation of three sites (purple dots). Compared with weaker CA3 activation in the lower panels, the stronger CA3 activation in upper panels causes more CA3 collateral activation and feedforward signal propagation to CA1. In later time points, CA3 activation also leads to some hilar activation.

The new aspects of our anatomical mapping results propelled us to confirm circuit connections to mossy cells and granule cells using functional/physiological mapping approaches. We first examined and compared EC functional inputs to DG granule and mossy cells using fast VSD imaging of neural activity and laser photostimulation via glutamate uncaging in brain slice preparations (Fig. 9*A*). In our VSD imaging experiments, the observed ensemble VSD signals are closely related to membrane potential depolarization of individual neurons (Xu et al., 2010a). This approach allows for high spatiotemporal-resolution imaging of the entire circuit, including the EC, DG, CA3, and CA1, and provides a macroscopic global view of evoked excitatory signal processes in the hippocampal formation.

Spatially restricted photostimulation in EC first initiates local cortical activation, and then excitatory signals propagate through the perforant path to evoke extensive activation in the DG blade with a short time latency (Fig. 9*B–E*). These observations are consistent across 5 different slice experiments using different animals. However, we find little activation in the hilus and CA3 in response to the EC stimulation at the image frames with initial DG activation (Fig. 9*B*,*C*). Our results support the idea that the DG functions as a gate at the entrance to the hippocampus, blocking or filtering incoming substantial excitation from the EC (Ang et al., 2006; Hsu, 2007). With stronger EC activation, DG allows some excitation to propagate to downstream regions including the hilus and CA3 (Fig. 9*D*,*E*). These physiologic data also support our anatomic data that EC does not directly or very weakly activate hilar mossy cells. The imaging experiments (*n* = 6) show that spatially restricted photostimulation of dentate granule cells activates hilar and CA3 responses, but hilar photostimulation does not result in intralaminar dentate granule activation (Fig. 9*F*). Furthermore, experiments (*n* = 5) show that CA3 photostimulation causes strong CA3 recurrent activation, and weakly evokes hilar responses. This demonstrates functional back projection of CA3 excitatory signals to the hilus (Fig. 9*G*).

**Figure 10.**
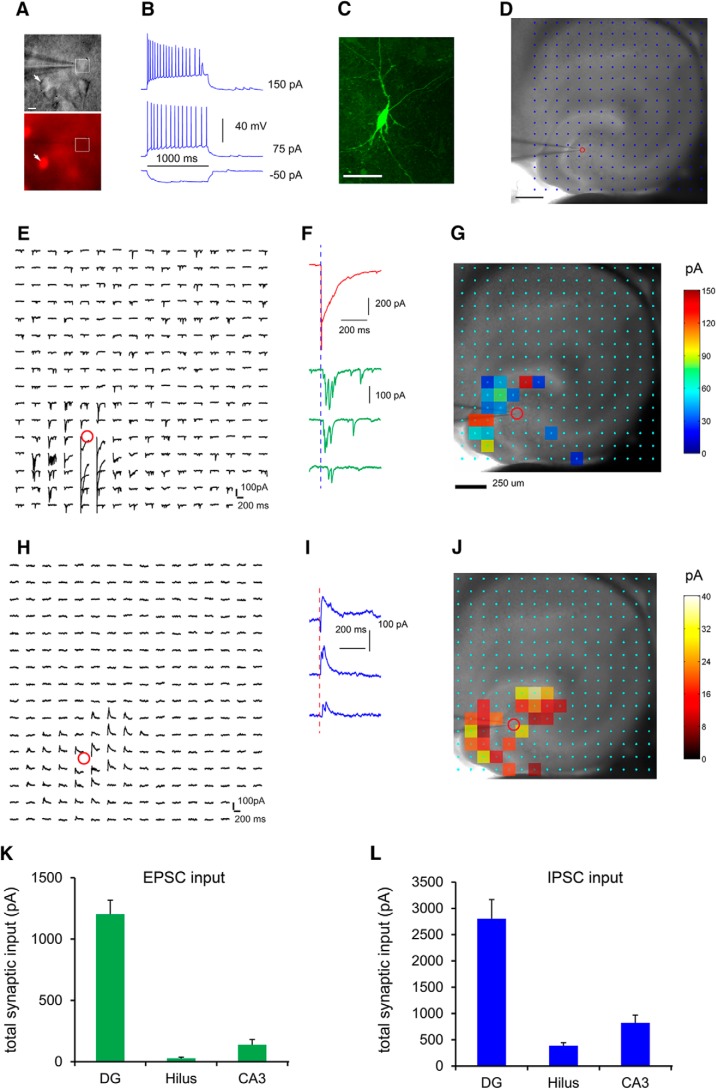
Local functional circuit mapping identifies strong DG versus weak CA3 inputs to hilar mossy cells. ***A***, Genetic label guided recordings of hilar mossy neurons in the horizontal hippocampal slices. Given that hilar mossy cells are the only glutamatergic cell type in the hilus, they are targeted by visualizing neurons with large somata and with nonred fluorescent expression (i.e., the white square in ***A***) in the dentate hilus of the Gad2-Cre; Ai9 mouse which expresses red fluorescent proteins (tdTomato) in GABAergic neurons (i.e., the arrowhead). ***B***, The targeted neuron in A shows regular adapting spiking in response to intrasomatic depolarizing current injection. ***C***, *Post hoc* identification of the recorded neuron as a mossy cell via intracellular biocytin staining. Scale bar, 50 μm. ***D–J***, LSPS allows for extensive and quantitative analysis of synaptic inputs to recorded mossy cells from local hippocampal circuits in a relatively large region. ***D***, A hippocampal slice image superimposed with photostimulation sites (cyan circles) spaced at 90 × 90 µm. The red circle indicates the tip of a recording electrode and the cell body location of the recorded mossy neuron. Scale bar, 200 µm. ***E***, The plot of EPSC responses from the recorded cell at the corresponding sites in response to photostimulation via glutamate uncaging. The response traces are plotted for 200 ms beginning at the photostimulation onset. ***F***, The two types of responses can be distinguished based on their waveforms, amplitudes, and response latencies. An example of direct uncaging responses is shown in red, and examples of synaptically mediated responses from presynaptic neuronal spiking are shown in green. The photostimulation (1 ms) is indicated by the vertical dashed blue line. The direct responses are excluded for synaptic input analysis. The raw data as shown in ***E*** are quantified and used for construction of a color-coded quantitative input map. ***G***, The input response sites are overlaid on the bright field image to show anatomic position. Scale bar, 250 μm. ***H***, The plot of IPSC responses from the recorded cell at the corresponding sites in response to photostimulation. The raw data as shown in ***H*** are quantified and used for construction of a color-coded quantitative input map. ***I***, Example IPSC responses from the recorded mossy cell. ***J***, The input response sites are overlaid on the bright field image to show anatomic position. ***K***, ***L***, Summary data of average total EPSC and IPSC inputs to the recorded mossy neurons measured from the DG granule cell and molecular layers (labeled as DG), hilus, and CA3.

Next, we combined whole cell recordings and LSPS to examine both local excitatory and inhibitory synaptic inputs to mossy cells in slice preparations in more detail (Fig. 10). Given that hilar mossy cells are the only glutamatergic cell type in the hilus, we used Gad2-Cre; Ai9 mice to aid our recordings of hilar mossy cells (Fig. 10*A*,*B*). In brain slices, we targeted neurons with large somata that lacked red fluorescent protein expression in the dentate hilus, excluding GABAergic interneurons (Fig. 10*A*). The recorded neurons were confirmed as mossy cells with intrinsic electrophysiology and *post hoc* morphologic analysis (Fig. 10*B*,*C*). This LSPS approach maps input sources across a relatively large region to single intracellularly recorded neurons, and provides a quantitative assessment of spatiotemporal distribution and input strength of excitatory and inhibitory connectivity to the recoded neurons (Fig. 10*D–J*). We performed region-specific analysis to characterize the excitatory input from different regions of hippocampus to mossy cells (*n* = 14 cells). Mossy cells receive strong excitatory afferent input from the DG (i.e., granule cells) with measurable input from CA3 excitatory neurons and very weak excitatory input from the hilus (i.e., other mossy cells; Fig. 10*E–G*). The average summed excitatory synaptic input strengths measured from the DG, CA3, and hilus are 1202.0 ± 115.4, 137.8 ± 43.1, and 26.3 ± 10.2 pA, respectively (Fig. 10*K*). Compared with rabies-tracing based whole brain measurements, the weaker functional readout of excitatory hilar input might reflect the inability of capturing extended mossy cell interconnections by *in vitro* slice mapping. Their inhibitory inputs are also from DG, CA3, and the hilus, with DG inhibitory inputs accounting for the bulk of total inputs (Fig. 10*H–J*). The average, summed inhibitory synaptic input strengths measured from the DG, CA3, and hilus are 2802.5 ± 365.9, 822.2 ± 146.8, and 386.3 ± 57.3 pA, respectively (Fig. 10*L*). Hence, the photostimulation-based circuit mapping supports VSD imaging results and further reveals both excitatory and inhibitory inputs from DG and CA3 to hilar mossy cells.

## Discussion

Mapping large-scale, direct synaptic inputs to hilar mossy cells and dentate granule cells is essential for understanding of how these cells are activated and function within hippocampal circuits. While hippocampal circuits have been systematically and extensively studied, for the first time we have provided an in-depth characterization of the local and long-range input connection projections to hilar mossy cells and dentate granule cells in quantitative and cell type-specific manners. This was made possible by the new viral tracing approach that enables quantitative evaluations of the numbers of targeted postsynaptic (starter) cells and their presynaptic cells labeled in local and long-range circuits. Using *in vivo* monosynaptic rabies tracing, we have uncovered previously unidentified and underappreciated circuit inputs to hilar mossy cells and dentate granule cells. We foresee that the new information derived from this study will be useful for guiding *in vivo* functional studies and quantitative DG circuit modeling analysis. Our novel combinatorial viral-genetic tracing and functional circuit mapping approaches can be extended to address other long standing questions in the hippocampus.

### Circuit connection comparisons between hilar mossy cells and dentate granule cells

We find that mossy cells receive highly convergent excitatory inputs from granule cells. This is supported by a previous study showing the viral label of commissurally projecting mossy cells and their afferent granule cells after injections of herpes simplex virus type 1 into the DG/CA3 area (Sík et al., 2006). The authors inferred that single mossy cells receive input from a compact cluster of 30-40 granule cells. Monosynaptic viral tracing indicates that mossy cells receive very little EC input, while DG granule cells receive very strong EC inputs. Given that hilar mossy cells receive a great majority of their excitatory and inhibitory inputs from the DG and CA3 circuits, it is reasonable to conclude that hilar mossy cells are the major local circuit integrators. Along with potential modulation by long range inputs from cholinergic MS-DB and purinergic SFi, mossy cells exert feedback modulation of activities of dentate granule cells and secondarily CA3 neurons once activated by strong DG/CA3 inputs.

In contrast to conventional notions of hippocampal circuit connectivity, our rabies tracing study finds that there are extensive, direct inputs from all CA3 subfields to dentate granule cells (also, see Vivar et al., 2012). Although the trisynaptic hippocampal circuit is believed to be strongly unidirectional, feedback pathways composed of both excitatory and inhibitory elements that relay information back from CA3 to DG should receive greater consideration. The functional implications in CA3-DG circuit operations suggest future studies to explore the role of feedback. The CA3 back-projection pathways to DG granule cells and hilar mossy cells are potentially organized in a complementary manner, as mossy cells receive stronger inputs from the proximal CA3 but granule cells receive stronger inputs from the distal CA3 (Figs. 1*M* and 5*M* ). In addition, there are more dorsal CA3 connections to targeted granule cells, but more mossy cell inputs from the ventral level to granule cells. These spatial distributions of CA3 and mossy cell inputs to granule cells may contribute to differential intralaminar and translaminar feedback modulation. Photostimulation-based circuit mapping via VSD imaging and whole cell recordings in brain slices in the present work and our published study (Shi et al., 2014) confirmed the direct back projection from CA3 to DG granule cells.

Spatially balanced inhibition and excitation seem to hold for both hilar mossy cells and DG granule cells. Hilar mossy cells receive inhibitory inputs parallel to excitatory inputs from within the DG and CA3. Similarly, DG granule cells receive local inhibition from the DG molecular layer and hilus, and CA3 as well. Generally, inhibitory input sources spatially matched those of excitatory inputs. In terms of circuit connection strengths measured by the CSI, hilar inputs (including both mossy cells and inhibitory interneurons) to DG granule cells are comparable to those of medial EC and CA3 excitatory neurons. It has been proposed that mossy cells mostly innervate hilar inhibitory neurons in the same lamina, and activation of mossy cells could lead to enhanced inhibition of the DG granule cells in the home laminae (Buckmaster and Schwartzkroin, 1994; Larimer and Strowbridge, 2008; Scharfman and Myers, 2012). This agrees with a recent study (Jinde et al., 2012), in which targeted ablation of mossy cells *in vivo* caused short-term hyperexcitability of granule cells using a transgenic mossy cell/CA3-Cre mouse crossed to a loxP-flanked diphtheria toxin receptor transgenic mouse. On the other hand, mossy cells send axons to primarily innervate DG granule cells across other laminae (Buckmaster et al., 1996; Wenzel et al., 1997). Our present study provides further anatomic evidence to support the granule cell association hypothesis (Buckmaster and Schwartzkroin, 1994; Scharfman and Myers, 2012).

In addition to their potential critical roles in cognitive functions of the DG, hilar mossy cells are interesting due to their vulnerability to excitotoxicity in temporal lobe epileptogenesis (Scharfman and Myers, 2012). Mossy cell loss has been observed both in humans with temporal lobe epilepsy and animal epilepsy models. The massive excitatory inputs from both DG and CA3 could put mossy cells at a high risk of loss in epileptic conditions, in which circuit inhibition is chronically dampened. Indeed, in slice and *in vivo* recordings, we and others have observed that mossy cells receive a great deal of excitatory drive from local circuits with a continuous barrage of large spontaneous EPSPs (Scharfman and Schwartzkroin, 1988; Scharfman, 1993; Buckmaster and Schwartzkroin, 1995; Strowbridge and Schwartzkroin, 1996). Interestingly, our circuit mapping reveals direct SFi input to mossy cells, but not DG granule cells. This might provide a new perspective on mossy cell vulnerability to excitotoxicity (Sperlágh et al., 2002).

### Technical considerations and future work

We think that a combined viral and mouse genetic approach can facilitate future studies of mossy cells. Jinde et al. (2012) made a transgenic mossy cell/CA3-Cre mouse, but specific genetic targeting of only hilar mossy cells is not possible yet using mouse genetics. In this study, we have followed the projection-based viral strategy (Gradinaru et al., 2010) to selectively target mossy cells. However, it might be more effective to perform spatially restricted viral targeting of mossy cells using the mossy cell/CA3-Cre mouse. This will overcome a potential caveat of the present study as the current strategy allows for contralaterally projecting hilar mossy cells to be targeted. Our tracing experiments would have missed the mossy cells that project only ipsilaterally, although no such “peculiar” mossy cells are known and this issue remains to be examined. In addition, it is important to complement the current work with new studies of anatomic and functional projections of targeted mossy cells. The same approaches we have developed can be similarly applied to examine various functional projections from these targeted cell types both *in vitro* and *in vivo*.

Finally, new and emerging techniques can help to answer questions of how mossy cells potentially integrate local circuit inputs and modulate DG-CA3 network activity, and what roles mossy cells may play in hippocampus-associated learning and memory behaviors (GoodSmith et al., 2017; Senzai and Buzsáki, 2017). Large scale neuronal population imaging of DG-CA3 circuit responses at single-cell resolution (Ziv et al., 2013; Danielson et al., 2017) in freely behaving animals, combined with viral genetic cell targeting, could elucidate how mossy neurons are activated *in vivo* and how they interact with DG and CA3 neurons to modulate place cell activities and ensemble representation of spatial and contextual environments.

## Synthesis

Reviewing Editor: Menno Witter, Norwegian University of Science and Technology Decisions are customarily a result of the Reviewing Editor and the peer reviewers coming together and discussing their recommendations until a consensus is reached. When revisions are invited, a fact-based synthesis statement explaining their decision and outlining what is needed to prepare a revision will be listed below.

This manuscript has been transferred from J Neurosci to eNeuro after revision. The revision and rebuttal indicate that the authors have managed to increase the quality of the manuscript. Although the statement of both reviewers concerning an overall low level of really new findings is still relevant, the paper provides new and well supported new findings.

There are a number of difficult sentences in the manuscript, some responses to reviewers are incomplete/inadequate and I request either deletion of figure 11 or an updated version. Please check the PDF file which I will email directly to you, which contains my comments and suggestions as well as a number of language corrections.
